# Cell-to-Cell Spread of Dengue Viral RNA in Mosquito Cells

**DOI:** 10.1155/2020/2452409

**Published:** 2020-06-25

**Authors:** Chih-Chieh Cheng, Chao-Fu Yang, Yin-Ping Lo, Yi-Hsuan Chiang, Eny Sofiyatun, Lian-Chen Wang, Wei-June Chen

**Affiliations:** ^1^Graduate Institute of Biomedical Science, College of Medicine, Chang Gung University, Kwei-San, Taoyuan 33332, Taiwan; ^2^Department of Public Health and Parasitology, College of Medicine, Chang Gung University, Kwei-San, Taoyuan 33332, Taiwan; ^3^Environmental Health Department, Banjarnegara Polytechnic, Central Java, Indonesia; ^4^Molecular Infectious Disease Research Center, Chang Gung Memorial Hospital, Kwei-San, 33305 Taoyuan, Taiwan

## Abstract

Dengue virus (DENV) is an important mosquito-borne arbovirus that is particularly prevalent in tropical and subtropical areas of the world. The virus is generally ingested with a blood meal, replicates in host tissues, and disseminates into salivary glands for transmission to the next host. Membrane-bound vacuoles carrying DENV particles have been documented in mosquito cells and play a role in the cell-to-cell transmission of DENV2. C189 is one member of the tetraspanin family and generally increases its expression as one component of the vacuoles (C189-VCs) within C6/36 cells infected with DENV2. In the present study, we have further demonstrated via sucrose gradient centrifugation as well as magnetic immune isolation (MI) that the RNA of DENV2 was eventually carried by C189-VCs. In addition, viral RNA was shown to spread from donor to recipient cells in a coculture assay even when 20 mM NH_4_Cl was added to inhibit virus replication in the culture. In an alternate assay using the transwell system, viral RNA was only detected in recipient cells in the absence of 40 mM NH_4_Cl, suggesting that cell-cell contact is required for the intercellular spread of DENV2. In turn, the formation of viral synapse (VS) derived from aggregates of viral particles was frequently observed at sites of cell contact. Taken together, the formation of C189-VCs in C6/36 cells is induced by DENV2 infection, which may serve as a vehicle for transferring virions and also viral RNA to neighboring cells by cell-to-cell transmission after cell-cell contact. This finding provides insight into the understanding of viral spread between mosquito cells. It may also elucidate the benign persistent infection in mosquito cells and efficient dissemination of DENV infection within a mosquito vector.

## 1. Introduction

Dengue virus (DENV) belongs to the family Flaviviridae [1]. The virus can be antigenically divided into four serotypes [2], each of which causes similar symptoms ranging from dengue fever (DF) with mild febrile illness to life-threatening dengue hemorrhagic fever (DHF) and dengue shock syndrome (DSS) [3]. According to a recent investigation, there are approximately 390 million dengue infections per year, of which 96 million manifest some level of disease severity [4]. Most outbreaks have been reported in tropical and subtropical regions [5]. In addition, at least 2.5~3 billion people are currently at risk of dengue infection in more than 100 countries, raising significant public health problems that are widely distributed globally [6, 7]. DENV is naturally transmitted between humans primarily by the mosquitos *Aedes aegypti* and *Aedes albopictus*, resulting in the establishment of outbreaks in endemic or epidemic areas [8, 9]. The spread of mosquito vectors is highly dependent on climate, population, and socioeconomic status [10], projecting dengue suitability and risk particularly into tropical areas [11].

As a mosquito-borne virus, DENV injected along with mosquito saliva after blood feeding generally infects Langerhans cells and keratocytes to initiate viral replication in the epidermis of a human bitten by the mosquito vector [12]. Human and other mammalian cells are usually infected by DENV through endocytosis mediated by receptor(s) that include dendritic cell-specific ICAM-grabbing nonintegrin (DC-SIGN), mannose, and C-type lectin domains containing 5A (CLEC5A) [13–15]. Susceptible mammalian cells infected by DENV mostly end up with apoptosis, leading to a large number of progeny virions bursting out from infected cells into the blood stream or cell culture to become the source of infection for other cells.

Like DENV, hepatitis C virus (HCV) is also a member of the Flaviviridae [16]. The chronic infection of HCV is generally established and maintained upon cell-to-cell transmission [17], which helps the virus evade host immunity [18]. There are two distinct modes extensively used by viruses for spreading among cells, one being the cell-free mode while the other executed by cell-to-cell transmission [19]. Cell-free virus spreading is generally very inefficient due to the existence of barriers located within donor or target cells [19]. In contrast, viral spread by cell-to-cell transmission will largely reduce the effects of those barriers and result in the rapid and efficient spread of the virus among cells. Human immunodeficiency virus (HIV) is known to transfer between CD4+ memory T cells via cell-to-cell transmission, resulting in a more efficient and rapid spread among cells [20]. Herpesviruses (HSV) also exhibit intercellular spread by cell-to-cell transmission with the advantage of evading host immunity [21]. The cell-to-cell transmission of DENV2 has been observed in mosquito cells, in which tetraspanin 189 was identified as being involved [22, 23].

The intestine of a mosquito is composed of a monolayer of epithelial cells resting on an extracellular basal lamina and is morphologically divided into the foregut, midgut, and hindgut [24]. The midgut is the site for temporarily storing a blood meal and where it is subsequently digested and absorbed. DENV ingested by mosquitoes with a blood meal generally initializes the scattered infection of epithelial cells in the midgut [25], followed by the formation of infection foci involving multiple cells. All the expanded foci may then merge to infect the entire organ within a few days of infection [26]. After replication, a large quantity of progeny DENV may accumulate in salivary glands before being transmitted to a human host [27].

Our previous observation has clearly shown that the tetraspanin C189 from C6/36 cells is upregulated in response to DENV2 infection, which was also found to colocalize with viral E protein in the cells [22]. When C6/36 cells were transfected with a C189-expressing construct, overexpressed C189 was incorporated into the membrane of virus-responsive vacuoles, called C189-containing membrane-bound vacuoles (C189-VCs), in which virions and/or viral proteins were confined [22]. Furthermore, we have also demonstrated that DENV2 virions can be transferred to neighboring cells via cell-to-cell transmission [23]. Apart from virions, more new evidence has shown that DENV RNAs can also spread efficiently in cells by the same route. This finding provides evidence to account for the successful dissemination of DENVs in a mosquito vector.

## 2. Materials and Methods

### 2.1. Cell Culture and Virus Propagation

The protocol for culturing C6/36 cells derived from the mosquito *Ae. albopictus* in this study followed a previously described method [28]. Briefly, the DENV2 virus (New Guinea C) was propagated in C6/36 cells grown in minimal essential medium (MEM) (Invitrogen, Carlsbad, CA) with nonessential amino acids containing 10 mM HEPES and 4.5 mM sodium bicarbonate and additional 10% fetal bovine serum (FBS) at 28°C in a closed incubator [22].

### 2.2. Plaque Assay

Virus titer determination was carried out by the plaque assay described in a previous report [28] on baby hamster kidney- (BHK-) 21 cells maintained at 37°C in an incubator with a 5% CO_2_ atmosphere.

### 2.3. Construction of the Expression Vector

The expression vector used in this study was constructed from the insect-cell expression vector pAC5.1-V5-His A (Invitrogen), following a previously established design for the expression of HA-C189. Briefly, primers HA-F (KpnI-HA-EcoRI-F: 5′-CATGTACCCATACGATGTTCCAGATTACGCTCG-3′) and HA-R (KpnI-HA-EcoRI-R: 5′-AATTCGAGCGTAATCTGGAACATCGTATGGGTACATGGTAC-3′) were hybridized and ligated to the pAC5.1-V5-His A to generate the pAC5.1-HA vector. Subsequently, the C189 gene was amplified using primers (forward: 5′-GCGCATCGAGAGGGAAAG-3′, and reverse: 5′-CATTGGTATGCGTTGATTCCAC-3′) and then inserted into the pAC5.1-HA to form the vector for HA-C189 expression.

### 2.4. Cell Transfection

Our cell transfection method followed the protocol previously described by this laboratory [23]. In brief, C6/36 cells were seeded into a 10 cm dish and grown to 70-80% confluence. Specific wells in the dish were transfected with MEM containing 10 *μ*g of pAC5.1-HA-C189 plasmid or the empty vector (control) by using 30 *μ*l FuGENE HP Transfection Reagent (Roche, Basel, Switzerland) for 5 h, followed by infection with DENV2 for 1 h. Subsequently, reagents were replaced with fresh culture medium and then incubated at 28°C for 24 h.

### 2.5. Detection of the Intercellular Spread of Viral RNA in the Transwell System

C6/36 cells (2 × 10^5^ cells/well) transfected with pAC5.1-eGFP were used as recipient cells and seeded onto the 6-well plate. Another batch of C6/36 cells was infected with DENV2 (MOI = 1) for 24 h and served as donor cells, which were then scratched off and transferred to the upper layer of the transwell system (24 mm insert with 0.4 *μ*m pore polycarbonate membrane) (Corning Incorporated Life Sciences, Tewksbury, MA, USA) after being washed five times with PBS. In this system, recipient and donor cells were separated but not limited for virus diffusion movement between layers. To scavenge released virus particles, the culture medium containing 40 mM NH_4_Cl was added and incubated at 28°C for 18 h. Those not treated with NH_4_Cl served as controls. For RNA identification, both donor and recipient cells were separately harvested from the plate and subjected to RNA extraction by using a GENEzol TriRNA Pure Kit (Geneaid Biotech, Taipei, Taiwan), followed by the protocols for reverse transcriptase-polymerase chain reaction (RT-PCR) mentioned below.

### 2.6. Detection of the Intercellular Spread of Viral RNA in the Coculture System

To establish DENV2-infected donor cells, C6/36 cells were infected with the virus at an MOI of 1 for 24 h. Another batch of C6/36 cells transfected with the eGFP expression vector was used as recipient cells. Donor cells washed with PBS to remove the cell-free virus were cocultured with recipient cells in the 40 mM NH_4_Cl medium (the ratio of donor cells to recipient cells was 1 : 1). At 18 h after coculture, cells positively expressing eGFP (recipient cells) were sorted out via a FACSAria IIU cell sorter (BD Biosciences, San Jose, CA, USA) and then subjected to RNA extraction. Viral RNA detection was carried out by RT-PCR as described below.

### 2.7. Cell Lysate Fractionation

In order to collect the cell lysate from C6/36 cells transfected with the plasmid pAC5.1-HA-C189, transfected cells were washed with PBS. Cells were scraped from the dish after adding 10% sucrose with lysis buffer (pH 7.4) containing 3 mM imidazole, 1 mM EDTA, RNase inhibitor (Sigma, St. Louis, MO, USA), and protease inhibitor cocktail (AG Scientific, San Diego, CA, USA). The cell lysate was then collected by disrupting cells using a 1 ml syringe with 27 G needle for at least 30 strokes, followed by centrifugation using a Microfuge® 22R microcentrifuge with F241.5P Rotor (Beckman Coulter, Atlanta, GA, USA) at 3500 rpm for 10 min. Post nuclear supernatant (PNS) collected after centrifugation was added to the top of a column containing 10-60% sucrose gradients containing 3 mM imidazole and 1 mM EDTA, from top to bottom. The column was then centrifuged in an Optima L-90K Ultracentrifuge with SW-41Ti Rotor (Beckman Coulter) at 41000 rpm at 4°C for 18 h. Subsequently, fractions containing 0.5 *μ*l were harvested from the top to bottom, with up to 23 fractions being collected for further analysis for RNA and protein.

### 2.8. Magnetic Immunoisolation

Cell lysate collected from C6/36 cells transfected with the plasmid pAC5.1-HA-C189 was centrifuged using a Microfuge 22R microcentrifuge (Beckman Coulter), and PNS was harvested for further magnetic immunoisolation by using a Dynabeads Protein G Immunoprecipitation Kit (Invitrogen). To prepare anti-HA Dynabeads, we added 50 *μ*l of them coated with protein G into the 8-tube Magnetic Separation Rack to remove the supernatant. Then, 100 *μ*l antibody binding and washing buffer containing 10 *μ*g mouse anti-HA IgG (Sigma) was added into the tube and gently shaken for 1 h. After another wash with antibody binding and washing buffer and centrifugation to remove the supernatant, prepared anti-HA Dynabeads were collected and subsequently mixed with PNS. After shaking at RT for 1 h, the mixture was transferred to an 8-tube Magnetic Separation Rack and washed three times with 200 *μ*l of washing buffer, followed by adding100 *μ*l of fresh washing buffer to resuspend the mixture and then moved into a new collection tube. For protein analysis, a mixture of 30 *μ*l 1x Laemmli sample buffer was heated in a water bath at 95°C for 5 min. Protein was then collected from the supernatant from the 8-tube Magnetic Separation Rack and subjected to Western blot analysis. For RNA detection, samples were RNA-extracted via a GENEzol TriRNA Pure Kit (Geneaid Biotech).

### 2.9. RNA Extraction

RNA extraction was carried out with a GENEzol TriRNA Pure Kit (Geneaid Biotech), following manufacturer instructions. In brief, 300 *μ*l samples collected from sucrose gradient centrifugation were mixed with 300 *μ*l GENEzol Reagent. After incubation for 5 min, 600 *μ*l of absolute ethanol was added. The mixture was subsequently transferred to an RB column set with a 2 ml collection tube, which was then centrifuged in a Microfuge 22R microcentrifuge (Beckman Coulter) at 14000 g for 1 min. Subsequently, a new 2 ml collection tube was substituted and 400 *μ*l of buffer added for a prewash and then centrifuged at 14000 g for 1 min. Another two washes were done with 600 *μ*l wash buffer and then centrifuged at 14000 g for 1 min, followed by the last 3 min centrifugation wash under the same conditions. A clean 1.5 ml microcentrifuge tube was then set up into the RB column in which 30 *μ*l DEPC-ddH_2_O was added. After 5 min of incubation, the column was centrifuged for 3 min at 14000 g. All collected samples were referred to RT-PCR analysis. For the magnetic immunoisolation samples, 5 × 10^6^ cells were added and mixed with 700 *μ*l GENEzol Reagent (Geneaid), but otherwise treated the same as those mentioned above.

### 2.10. Reverse Transcriptase-Polymerase Chain Reaction (RT-PCR)

Extracted total RNA in this study was used as the template, from which first-strand cDNA was synthesized via reverse transcriptase by using a SuperScript First-Strand Synthesis kit (Invitrogen) following manufacturer instructions. The viral RNA level in DENV-infected cells was subsequently detected by the application of synthesized cDNA and the primer pair located at the 5′-UTR, including DV2-1F (5′-TGGACCGACAAAGACAGATTC-3′) and DV2-1R (5′-CATGTGTGGTTCTCCGTTACG-3′). The internal control gene 18S was detected with primers 18S-F (5′-TGACTCAACACGGGAAAAC-3′) and 18S-R (5′-CAGAACATCTAAGGGCATCAC-3′). The predicted PCR product sizes were 459 bp and 358 bp for the virus and 18S gene, respectively. The C189 expression level was normalized to the 18S expression level. To identify strand-specific viral RNA, cDNA was synthesized using the DV2-1F primer for the positive strand while the DV2-1R was used for the negative strand. Specific strands of viral RNA were then amplified with the primer pair consisting of DV2-N1F (5′-CTGAAACGCGAGAGAAACCG-3′) and DV2-N1R (5′-GTATCCCTGCTGTTGGTGGG-3′).

### 2.11. Western Blot

Protein harvested from C6/36 cells that were either infected by DENV2 or not was boiled for 3 min, then separated by electrophoresis on 12% (*w*/*v*) sodium dodecyl sulfate polyacrylamide gel (SDS-PAGE) in nonreducing conditions. It was subsequently transferred onto an Immobilon-P Transfer Membrane (Millipore, Darmstadt, Germany). After blocking with 5% milk-TBS-0.1% Tween 20 buffer at RT for 1 h, the membrane was incubated with the indicated primary and secondary antibodies at RT for 1 h as the method done previously in this lab [29]. Specific primary antibodies included the 4G2 monoclonal antibody (a kind gift of Dr. Guey-Chuen Perng, National Cheng Kung University, Taiwan) for the dengue E protein and anti-actin mouse monoclonal antibody clone C4 (Merck Millipore, Burlington, MA, USA). Secondary antibodies were goat anti-rabbit or anti-mouse IgG antibodies, depending on the primary antibody used in the experiment. After the final wash, the membrane was treated with a Western Lightning Chemiluminescence Plus Reagent (PerkinElmer, Waltham, MA, USA), from which signals were detected on a FUJI X-ray film.

### 2.12. Transmission Electron Microscopy with In Situ Embedding

Electron microscopy used in this study followed a previously described method [28]. Briefly, C6/36 cells seeded on the dish were immediately fixed with a mixture of 2% (*v*/*v*) glutaraldehyde and 2% paraformaldehyde in 0.1 M cacodylate buffer (pH 7.4) overnight at 4°C. After cells were postfixed in 1% (*w*/*v*) osmium tetroxide in 0.1 M cacodylate buffer for 2 h at room temperature, they were washed with 0.2 M cacodylate buffer three times. Again, cells were washed with 0.2 M cacodylate buffer three times and then dehydrated through an ascending graded series of ethanol. Cells were embedded *in situ* with Spurr's resin (Electron Microscopy Science, Hatfield, PA, USA), followed by polymerization at 70°C for 72 h. Trimmed blocks were sectioned with an ultramicrotome (Reichert Ultracut R, Leica, Vienna, Austria), and the ultrathin sections were stained with saturated uranyl acetate in 50% ethanol and 0.08% lead citrate in sequence. Selected images were observed and photographed under a transmission electron microscope (JEOL JEM-1230, Tokyo, Japan) at 100 kV.

## 3. Results

### 3.1. Confirmation of DENV Colocalized with Transfected C189 in C6/36 Cells

**Figure 1:**
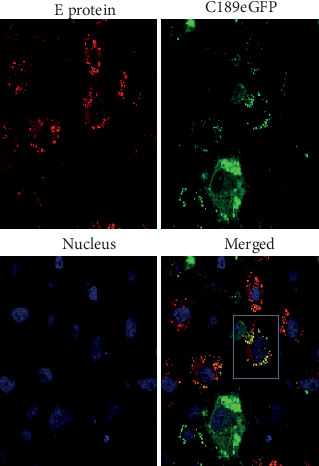
Confirmation of DENV colocalized with transfected C189 in C6/36 cells. In DENV2-infected C6/36 cells transfected with eGFP-tagged expressing vector containing the C189 insert, E protein was observed to colocalize with overexpressed C189 as shown in the merged image at 24 h postinfection. Red: DENV E protein; green: eGFP-tagged C189; blue: DAPI-stained nucleus. Images are shown under a laser scanning confocal microscope. Original magnification: ×100.

During the transfection of an eGFP-tagged expressing vector containing C189 that was inserted into DENV2-infected C6/36 cells, viral E protein was detected in a close localization with overexpressed C189 (Figure 1). This confirmed that C189, which is usually elicited by DENV2 in C6/36 cells, is distributed along with progeny virions within infected cells. Virions may be mostly contained within C189-containing vacuoles (C189-VCs) [23].

### 3.2. Identification of Viral Components from Stratified Cell Lysate

**Figure 2:**
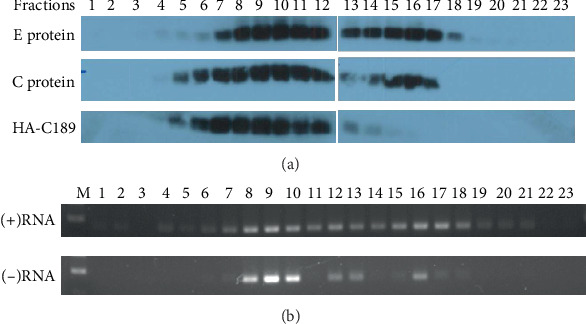
Identification of DENV RNAs and C189 in stratified fractions of cell lysate by sucrose centrifugation. (a) Viral proteins and RNA were detected from fractions collected by sucrose centrifugation from DENV2-infected C6/36 cells transfected with the C189-overexpressing vector and infected by DENV2. A total of 23 fractions were collected in top-to-bottom order, from which viral E protein was identified from fractions 7~17 while C protein appeared in fractions 5~17 and C189 was detected mainly from fractions 5~12. This implied that progeny virions actually exist and colocalize with C189. (b) Viral RNAs, including positive- (+) or negative- (-) sense, were detected from the selected fractions (7~18 for the positive sense and 8~16 for the negative sense). In particular, (-) RNA was identified in fractions 8~10 in parallel with the distributions of viral E and C proteins and C189. This shows that DENV2 virions, proteins, and RNAs are contained in virus-induced C189-VCs. M: DNA markers.

In order to differentiate the distributions of viral proteins, RNA, and induced C189, 23 fractions were selected in order from the top to the bottom of the lysate of C6/36 cells transfected with C189-overexpressing vectors and infected by DENV2 (Figure 2(a)). Viral E protein was mainly identified from fractions 7~17, while C proteins appeared in fractions 5~17 and C189 was clearly detected from fractions 5~12 (and may extended to fraction 14). It is apparent that there is a parallel distribution between them, implying that variable numbers of progeny virions may accompany C189.

More significantly, either positive- (+) or negative- (-) sense RNAs of the virus were also detected from the cell lysate of selected fractions (fractions 7~18 for the positive sense and 8~16 for the negative sense) (Figure 2(b)). (-) RNA was mostly detected from fractions 8~10, which is also where viral E and C proteins and C189 were distributed. This concurrent distribution further confirmed that DENV2 and its components (including virions, proteins, and RNAs) are colocalized with or contained within the induced C189-VCs.

### 3.3. Detection of Viral RNA from C189-VCs Collected by Immunoisolation

**Figure 3:**
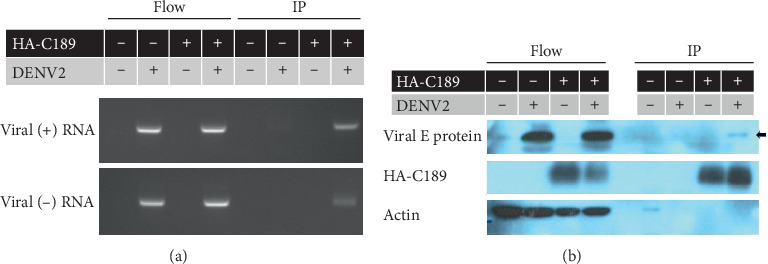
Detection of viral RNA and E proteins from immunoisolated C189-VCs collected from DENV2-infected C6/36 cells. (a) C189-VCs were collected from C6/36 cells (DENV2-infected and C189-transfected) via immunoisolation. Subsequently, both (+) and (-) viral RNAs were detected in the flow sample; i.e., unprecipitated cell lysates. Both strands of viral RNA were identified from immunoisolated cell components (IP), suggesting that C189-VCs actually contain viral RNA, both (+) and (-), during DENV2 infection in C6/36 cells. (b) The Western blot result showed that viral E protein can be detected from IP (arrow), further indicating that virions and/or viral components are contained within C189-VCs.

Through immunoisolation using HA antibody-binding Dynabeads, C189-VCs were collected from DENV2-infected C6/36 cells and then used for the detection of viral RNA. Both (+) and (-) viral RNAs were detected in the flow sample, i.e., unprecipitated cell lysates, as expected (Figure 3(a)). In addition, both strands of viral RNA were clearly identified from immunoisolated cell components, suggesting that C189-VCs eventually carry viral RNA, both (+) and (-), during DENV infection in C6/36 cells. The existence of (-) sense RNA also indicated that DENV replicates during infection in C6/36 cells, very likely occurring in C189-VCs. According to the Western blot result, the viral E protein can also be detected from IP (Figure 3(b)). It further indicated that virions or viral components are contained within C189-VCs in addition to viral RNA as mentioned above.

### 3.4. Delivery of Viral RNA from Donor to Recipient Cells

**Figure 4:**
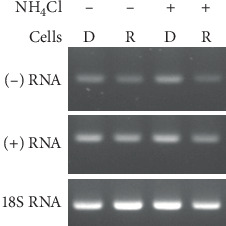
Delivery of viral RNA from donor to recipient cells in the coculture system. The intercellular delivery of viral RNA was investigated in the coculture system containing infected (donor, or D) and uninfected (recipient, or R) cells. During the culture, 40 mM NH_4_Cl was used to inhibit the infection of recipient cells by cell-free DENV2 released from donor cells. The results showed that both (+) and (-) viral RNAs were detected from recipient cells even though 40 mM NH_4_Cl was applied to the culture, suggesting that cell-to-cell transmission may have occurred in this culture.

**Figure 5:**
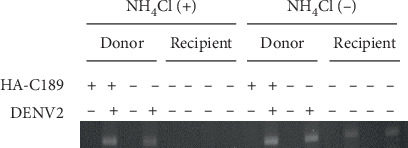
Delivery of viral RNA from donor to recipient cells in the transwell system. Intercellular delivery of viral RNA was also investigated in the transwell system containing infected (donor) and uninfected (recipient) cells. DENV (-) RNA was detected only in donor cells, either treated with 40 mM NH_4_Cl or not, whether they were transfected with HA-C189 or not. On the other hand, viral RNA was identified from recipient cells only in the absence of 40 mM NH_4_Cl treatment, further suggesting that DENV2 can be delivered from donor to recipient cells upon cell-cell contact.

In order to see the intercellular trafficking of viral components, 40 mM NH_4_Cl was used to inhibit the infection of recipient cells by cell-free DENV2 released from donor cells. When donor cells were cocultured with recipient cells, both (+) and (-) viral RNA were clearly detected from recipient cells even though 40 mM NH_4_Cl was applied to the culture system (Figure 4). In the meantime, using the transwell system, (-) viral RNA was detected in uninfected recipient cells only in the absence of 40 mM NH_4_Cl (Figure 5). Nevertheless, RNA was detected in donor cells, either treated with 40 mM NH_4_Cl or not, even though they had been transfected with HA-C189 (Figure 5). This suggests that DENV2 may be delivered from donor cells to recipient cells upon cell-cell contact, which is required for cell-to-cell transmission.

### 3.5. Viral Synapse Formation at the Site of Cell-Cell Contact

**Figure 6:**
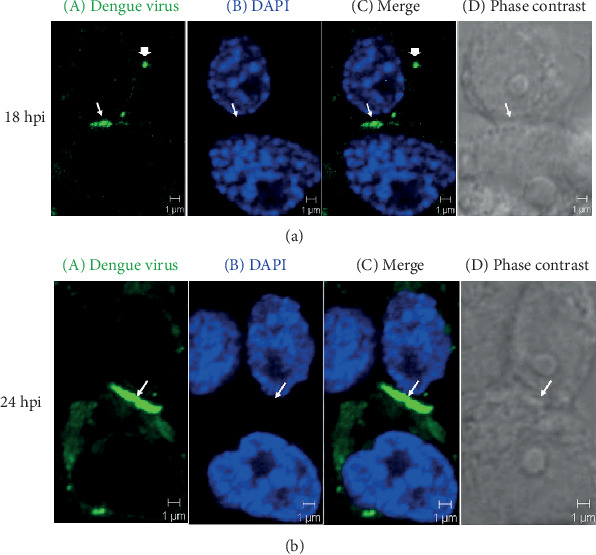
Viral synapse formation at the site of cell-cell contact. The results of DENV E protein detection by immunofluorescent assay (IFA) revealed that viral products may accumulate at the site of cell contact (arrow) at 18 hpi (a) and form an evident viral synapse (arrow) at 24 hpi (b). Green: DENV E protein; blue: DAPI-stained nucleus.

**Figure 7:**
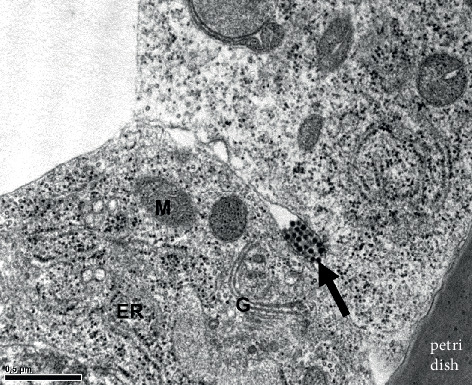
Virion aggregates in the space between cells at the ultrastructural level. Based on observations by transmission electron microscopy, virions were shown to concentrate in the space between cells (arrow), supposedly the location of VS in C6/36 cells at 24 hpi. ER: endoplasmic reticulum; G: Golgi apparatus; M: mitochondria. Scale bar = 0.5 *μ*m.

The detection of virions with E protein antibody by IFA on C6/36 cells infected by DENV2 revealed that virions frequently aggregated in intercellular spaces (i.e., the sites of cell-cell contact) in addition to the cytoplasm. The accumulation of virions usually occurred in the spaces between cells starting at 18 hpi (Figure 6(a)), followed by the appearance of higher concentrations of viral aggregates and formation of the “viral synapse (VS)” at 24 hpi (Figure 6(b)). At the ultrastructural level, aggregates of virions were also observed in intercellular spaces, supposedly the VS, at 24 hpi (Figure 7). This implies that cell-cell contact might be necessary for successful cell-to-cell transmission, facilitating the delivery of virions and other viral components from an infected cell to its neighbors.

## 4. Discussion

Being a member of the mosquito-borne arboviruses, DENV requires the infection of mammalian and mosquito cells in its natural cycle. Generally, the virus infection in the mosquito disseminates from the initial site of infection (i.e., the midgut) to the salivary glands after a few days of incubation before it can be transmitted to humans [27]. During DENV replication in the mosquito, the virus accompanying ingested blood meals will initiate infection in a few individual epithelial cells of the midgut, forming multiple small-sized infection foci and then extending throughout the epithelium of the midgut [26]. It is interesting to see how an efficient dissemination of DENV occurs in the midgut and other tissues in an infected mosquito. In culture, DENV infects mosquito cells generally through a mode of release and entry or infection by cell-free virus. Nevertheless, the spread of DENV in the midgut is probably not dependent in the same way, since the mosquito midgut is structurally composed of a monolayer of epithelial cells that strictly limit the space available [30]. In addition, most mosquito cells are able to survive DENV infection for a longer period of time through a combination of host defenses [28, 31]. Consequently, this results in persistent infection in many instances [32]. Rather recently, the cell-to-cell transmission of DENV2 has been documented as an alternative route for the spreading of progeny virions in C6/36 cells [23]. In combination with these observations, a resultant benign infection of DENV may be sustained lifelong within its mosquito vectors.

Most plant viruses move from one cell to another to establish infection, indicating that cell-to-cell transmission of the virus is important during the infection [33]. In fact, cell-to-cell transmission is increasingly shown to occur in many kinds of animal viruses by taking advantage of immune evasion [34]. Human immunodeficiency virus type 1 (HIV-1) and human T-lymphotropic virus type 1 (HTLV-1) are examples, showing a higher infection rate in dendritic cells via cell-to-cell transmission compared to cell-free viruses [35]. For most viral infections in host tissues, this mode of cell-to-cell transmission is more advantageous than the process of release and entry between cells, with the advantages of rapidity and efficiency in dissemination [34]. It may also result in the long-distance movement of the virus. In HCV chronic infection, cell-to-cell transmission is critical since it favors the escape of host neutralization, and the virus thus becomes resistant to direct-acting antiviral agents [36]. In addition, cell-to-cell transmission helps infected cells resist host innate immunity and thus creates an environment favoring the survival of infected cells [37]. In some cases, cell-to-cell transmission may promote infection in cells that do not have the corresponding receptor(s) [38].

Very importantly, viral components other than virions including proteins and even genomic material may also be transferred from infected to uninfected cells via cell-to-cell transmission. The viral RNA in plants commonly uses this transmission route, which could help the virus overcome protective cellular barriers between cells [33]. This fast lane for spreading viruses in cells has been frequently observed in various animal viruses [38], facilitating the establishment of rapid and efficient infection in targeted cells. More specifically, the cell-to-cell transmission of viral RNA is extensively documented, particularly in viruses with positive-sense RNA genomes such as the bovine viral diarrhea virus [39]. Generally, the transfer of genomic viral RNA occurs upon the stochastic transfer of genomic RNAs into neighboring cells [17]. This inherently random process provides an equal opportunity for each targeted cell to receive beneficial RNAs from infected neighboring cells [40]. Eventually, short fragments of DENV RNA can be recovered from patient sera, implying that sections of the parental genome may be deleted during infection [41]. It turns out that the virus benefits by retaining its intact genome in progeny populations during cell-to-cell transmission of its RNA. Ultimately, it accelerates viral adaption during its evolution [42].

Usually, viruses need an inherent spatial process for spreading within the host before further transmission to other hosts [43]. Direct cell-to-cell transmission is undoubtedly an efficient mode for spreading the virus, resulting in maintaining a persistent infection within the host [17]. It seems particularly suited for DENV infection within the mosquito vector, in which the infection of multiple tissues via dissemination from the midgut to salivary glands is needed [26]. The present results reveal that the cell-to-cell transmission of DEN viral RNAs, in addition to virions, eventually occurs in mosquito cells. This transmission route is not only rapid for dissemination but also equally beneficial for the spread of intact viral RNA that may be formed during replication.

Taken together, DENV in C6/36 cells may spread its infection by the mode of cell-to-cell transmission. With the assistance of C189-VCs formed in response to the infection, virions as well as viral proteins and RNA can be efficiently transferred from infected into uninfected cells. As cell contact is required during the process of cell-to-cell transmission [23], the formation of VS derived from high concentrations of viral particles was frequently observed at sites of cell contact. In addition to DENV, this feature has been observed in the cell-to-cell transmission of HIV in T cells [44, 45]. As reported, DENV-infected mosquito cells, either the donor cell or the target (recipient) cell, mostly remain undamaged during the infection [32, 34]. The present finding, in addition to the intercellular transmission, also provides a deeper insight into the mechanism of lifelong benign and persistent infection sustained in the mosquito vector infected by the DENV.

## Data Availability

The data used to support the findings of this study are included in the article.
